# PCSK6 is a novel regulator of venous smooth muscle cell function in arteriovenous fistula remodeling

**DOI:** 10.1080/0886022X.2026.2663246

**Published:** 2026-05-05

**Authors:** Xiangjiang Guo, Ruzhou Cao, Tianchen Wang, Lan Zhang, Yinan Li

**Affiliations:** a Department of Vascular Surgery, Renji Hospital, Shanghai Jiao Tong University School of Medicine, Shanghai, China; b Department of Vascular Surgery, Ningbo Hangzhou Bay Hospital, Ningbo, China; c Department of Biomedical Engineering, Whiting School of Engineering, Johns Hopkins University, Baltimore, USA

**Keywords:** Arteriovenous fistula, PCSK6, vascular remodeling, STAT1

## Abstract

The arteriovenous fistula (AVF), the preferred vascular access for hemodialysis, is limited by neointimal hyperplasia. Although proprotein convertase subtilisin/kexin type 6 (PCSK6) is involved in vascular smooth muscle cell (VSMC) activation, its role in AVF stenosis is unclear. PCSK6 expression was significantly upregulated in VSMCs of human and murine stenotic AVFs and correlated with the severity of neointimal hyperplasia. *In vitro*, PCSK6 overexpression promoted VSMC proliferation, migration, contractility, and extracellular matrix (ECM) synthesis, whereas PCSK6 knockdown suppressed these processes. Mechanistically, PCSK6 directly interacted with STAT1 and enhanced its phosphorylation, with STAT1 inhibition reversing PCSK6-driven effects. *In vivo*, smooth muscle cell-specific PCSK6 knockout in mice on a high-glucose diet attenuated neointimal formation, improved AVF lumen diameter and blood flow, and enhanced vasodilation. In conclusion, PCSK6 drives AVF neointimal hyperplasia by binding and activating STAT1 to promote VSMC phenotypic switching and ECM remodeling, identifying the PCSK6/STAT1 axis as a novel mechanism and potential therapeutic target for preventing AVF failure.

## Introduction

End-stage renal disease (ESRD) is a condition that primarily relies on renal replacement therapy, with hemodialysis being a primary treatment modality [1]. A functional vascular access is crucial for hemodialysis efficacy, with the arteriovenous fistula (AVF) representing the preferred modality due to its superior patency and lower complication rates compared to arteriovenous grafts and central venous catheters [2]. Nevertheless, up to 50% of AVFs fail to mature adequately for routine dialysis [3,4], primarily due to venous neointimal hyperplasia, which is characterized by excessive cellular proliferation and extracellular matrix (ECM) deposition within the venous wall, leading to stenosis and eventual access failure [5–7].

Accumulating evidence indicates that phenotypic switching of vascular smooth muscle cells (VSMCs) plays a pivotal role in neointimal hyperplasia pathogenesis [8–10]. In response to hemodynamic stress and local inflammation, VSMCs undergo dedifferentiation from a contractile to a synthetic state, accompanied by increased proliferation, migration, and secretory activity [9,11]. This transition promotes neointimal expansion and vascular remodeling, yet the underlying regulatory mechanisms remain incompletely elucidated. Previous research has found that the synthetic-state VSMCs exhibit enhanced proliferative capacity [12,13], enabling their rapid expansion within the intimal layer. Concurrently, their migratory potential is upregulated, facilitating their translocation from the medial layer to the intima [14]. Furthermore, these cells secrete abundant extracellular matrix (ECM) components, such as collagens and fibronectin, which constitute a major part of the expanding neointimal lesion. Collectively, these processes, which are driven by the phenotypically switched VSMCs, directly contribute to neointimal thickening and pathological vascular remodeling [15].

Recent studies identified PCSK6 as one of the most significantly upregulated molecules in human atherosclerotic plaques [16], while its function in AVF remodeling remains unexplored, which prompted us to investigate its potential involvement in AVF stenosis.

In this study, we report that PCSK6 expression is markedly elevated in VSMCs of human and murine stenotic AVFs and correlates with the severity of neointimal hyperplasia. Through integrated gain- and loss-of-function experiments *in vitro* and smooth muscle-specific PCSK6 knockout models *in vivo*, we demonstrate that PCSK6 drives VSMC proliferation, migration, contractility, ECM synthesis, and MMPs activation. Furthermore, we identify STAT1 as a direct binding partner and downstream effector of PCSK6 signaling. Our results establish the PCSK6/STAT1 axis as a novel regulatory mechanism in AVF neointimal hyperplasia, offering potential therapeutic targets for improving vascular access patency in ESRD patients.

## Material and methods

### Patient tissue collection

The collection and use of human biological samples (human arteriovenous fistula tissues) in this study were carried out in accordance with the Declaration of Helsinki (2024) and approved by the research ethics committee of the Renji Hospital (KY2024-139-B). All patients included in this research provided written informed consent. Human arteriovenous fistula (AVF) tissues were harvested from patients undergoing surgical closure of AVF combined with vascular reconstruction. Preoperatively, the ultrasonic diagnostic threshold for the stenotic group was defined as a luminal stenosis rate of ≥50%, and patients without detectable AVF vascular stenosis were assigned to the non-stenotic group. The detailed surgical procedure was performed as follows: the skin and subcutaneous fat were incised layer by layer to expose the arteriovenous anastomosis, arterial segment, and venous segment of the artificial AVF. Small atraumatic vascular clamps were gently applied to occlude the proximal arterial segment, distal arterial segment, and proximal venous segment of the anastomosis, respectively. The anastomotic tissue of the artificial fistula was precisely resected along the anastomotic margin with microvascular scissors, with the intact normal vascular walls of the artery and vein preserved. The cephalic vein stump was trimmed, and the lateral arterial orifice was repaired with a continuous suture technique. The cephalic vein stump, after its separation from the radial artery, was completely ligated with a double ligation and transfixion suture to achieve permanent closure of the abandoned artificial AVF and eliminate the abnormal blood flow pathway. Specifically, the stump was initially ligated with 4-0 silk sutures, followed by a transfixion suture at the proximal side of the ligation line. AVF tissue samples from patients in different groups were collected during this surgical procedure. The donor patients were matched for age, sex, duration of prior hemodialysis, and time since transplantation. Preoperative ultrasonography was performed to measure AVF blood flow and assess for stenosis. We explicitly excluded patients complicated with peripheral arterial disease, autoimmune diseases, diabetic angiopathy, and coagulation dysfunction. A total of 12 AVF tissue samples, comprising both stenotic (*n* = 6) and non-stenotic (*n* = 6) specimens, were collected. The collected tissues were fixed in 4% paraformaldehyde for 24 h and subsequently embedded in paraffin or snap-frozen in liquid nitrogen for subsequent analysis. All participants provided written informed consent. Detailed patient characteristics are provided in supplementary Table 1. The 12 enrolled patients with AVF, comprising 6 with stenosis and 6 without stenosis, showed no significant differences in core clinical indicators following grouped comparisons and statistical analysis of baseline characteristics (Figure S1).

#### Animal models of AVF

The animal protocols used in this study were approved by the Ethics Committee of Renji Hospital (RJ2019-0320), and the study was conducted following the National Institutes of Health Guidelines for the Care and Use of Laboratory Animals (SYXK-2018-0013). For the animal studies, all the C57BL/6 mice were male. All mice were housed under pathogen-free conditions at a temperature of 23 °C under a 12-h dark/light cycle. To promote stenosis development, a high-glucose diet was given with 45% kcal glucose (purchased from Jiangsu Medisen Biomedical Co., Ltd.), which was initiated immediately after the completion of AVF surgery and continued for 8 weeks until the experimental endpoint. The food intake and feeding environment of all mice were kept consistent. After genotyping and confirmation of inclusion criteria, animals were allocated to experimental or control groups by simple randomization. To minimize potential confounding by time or operator, the order of surgeries and outcome assessments was alternated between groups. To reduce environmental bias, cage positions were rotated weekly. All collected tissues were processed for subsequent RNA-seq analysis. Total RNA from snap-frozen tissue was isolated using the mirVana Isolation Kit (Ambion Cat#:AM1560). RNA sequencing was performed on Illumina HiSeq 2000 according to standard protocols.

The smooth muscle cell-specific conditional knockout of the mouse *Pcsk6* gene was created employing CRISPR/Cas9 and Cre-loxP systems. Exons 3–5 will be selected as the conditional knockout region (cKO region). The region contains a 326 bp coding sequence. The corresponding control group consisted of PCSK6 wild-type mice (pcsk6WT). Experimental units were defined as entire litters in which all pups carried the same genotype, either Pcsk6 cKO or pcsk6WT control.

Deletion of this region should result in the loss of function of the mouse *Pcsk6* gene. Two sgRNAs targeting were designed using the CRISPR design tool. These sgRNA sequences were synthesized and inserted into the pX330 vector containing the Cas9 nuclease gene. The Cas9 and sgRNA sequences were then incorporated into a loxP-flanked vector (floxed Cas9/sgRNA vector), enabling Cre recombinase-dependent activation of Cas9 and sgRNA expression. Single-cell stage mouse zygotes were microinjected with this floxed Cas9/sgRNA vector and transferred to pseudopregnant mice to generate transgenic offspring. PCR screening of offspring genomic DNA identified mice carrying the floxed Cas9/sgRNA construct. *Tagln*-CreERT2 mice, which express Cre recombinase under an endothelial-specific promoter activated by tamoxifen, were chosen for breeding. Floxed Cas9/sgRNA transgenic mice were mated with CreERT2 mice to obtain offspring with endothelial-specific Cre expression. Double-positive offspring (floxed Cas9/sgRNA and *Tagln*-CreERT2) received tamoxifen (100 μg/g, intraperitoneal) daily for five days to induce Cre recombinase activity specifically in endothelial cells. Following tamoxifen treatment, smooth muscle cells were isolated. Genomic DNA was extracted, and exon IIIc region PCR amplification with subsequent sequencing verified exon IIIc deletion.

Mice aged 9–12 weeks underwent surgical creation of infrarenal AVF. Furthermore, we also included a sham-operated control group where mice underwent laparotomy and vascular dissection without fistula creation. Only male mice were studied since female sex is the only predictor of non-maturation of human AVF in some studies. Based on prior murine arteriovenous fistula (AVF) studies and pilot data, the experimental cohorts comprised nine Pcsk6 conditional knockout (cKO) mice and nine Pcsk6 wild-type (WT) mice, totaling 18 animals. Briefly, AVF were created by needle puncture from the aorta into the inferior vena cava (IVC) using a 25 G needle. A standardized puncture depth of 2 mm was adopted, and the needle was kept for 10s after puncture and then slowly withdrawn to ensure a consistent fistula diameter (about 0.25 mm) in all mice. Meanwhile, the surgery was performed by a single senior experimenter to minimize the model inconsistency caused by operational differences. Visualization of pulsatile arterial blood flow in the IVC was assessed as a technically successful creation of AVF. Following surgery, all animals were monitored daily and evaluated weekly by a veterinarian for changes in health status. Doppler ultrasound (40 MHz) was used to confirm the patency of the AVF and to measure the diameter of the vessels. Mice were positioned on the operation panel facing upward and were placed under anesthesia with isoflurane. Hair on the abdomen was removed using depilatory cream. Ultrasound images of the aortas were obtained using a Vevo 2100 high-resolution imaging system. The probe was first applied on the long axis to seek out the abdominal aorta to record morphological images and then moved to the short axis to measure the maximum aortic diameter. Color mode Doppler was then activated and used to localize the flow around 6 renal arteries. To minimize bias, postoperative follow-up and ultrasound imaging were performed by investigators blinded to group allocation.

Euthanasia of mice was performed in accordance with the guidelines of the American Veterinary Medical Association (AVMA) Panel on Euthanasia (2020). Specifically, mice were placed in a closed, well-ventilated anesthesia chamber connected to an isoflurane vaporizer (VetEquip, Inc., Pleasanton, CA, USA) and an oxygen source. Isoflurane (Baxter Healthcare Corporation, Deerfield, IL, USA) was administered at an induction concentration of 3–5% (v/v) in oxygen (flow rate: 1 L/min) until the mice, assessed by the absence of a righting reflex and response to a toe pinch lost consciousness. The isoflurane concentration was then maintained at 2-3% (v/v) for an additional 5–10 min, until cessation of spontaneous respiration and heartbeat, which was monitored *via* visual observation of chest movement and tactile pulse detection at the femoral artery. To ensure irreversible death and avoid potential recovery, a secondary physical method was performed immediately after the confirmation of respiratory/cardiac arrest. All procedures were conducted by trained personnel to minimize animal distress.

### Immunohistochemistry and immunofluorescence

After euthanasia, the circulatory system was flushed under pressure with PBS followed by 10% formalin, and the AVF was harvested en bloc. The tissue was then embedded in paraffin and cut in 5 μm cross sections. Hematoxylin and eosin staining was performed for all samples. Six equidistant points around the IVC and opposite the aortic wall were averaged in each cross section to obtain the mean AVF outer wall thickness. Immunofluorescence staining was performed on tissues fixed in 4% paraformaldehyde. After permeabilization with 0.25% Triton X-100, samples were blocked with 1% BSA for 1 h at room temperature. Primary antibody incubation was followed by PBS washes containing 0.1% Triton X-100, then incubation with FITC-conjugated secondary antibody (diluted 1:1000 in PBST with 1% BSA). Images were acquired using the Zeiss fluorescence microscope. Complete antibody details are available in Supplementary Table 2.

#### Vasomotor responses

AVF relaxation assays were performed using acetylcholine (Ach, Sigma-Aldrich). AVF rings (4 mm in length) were excised and immediately placed in ice-cold Krebs buffer. The rings were then mounted on hooks and suspended in 10 mL tissue chambers containing oxygenated (95% O_2_/5% CO_2_) Krebs buffer maintained at 37 °C. The Krebs buffer in the chambers was refreshed every 15 min. Following a 60-min equilibration period, the rings were pre-constricted with 3 × 10^−6^M phenylephrine until a stable contraction plateau was achieved. Subsequently, cumulative dose-response curves were constructed by sequential administration of ACh. Changes in tension were recorded continuously using an isometric force-displacement transducer. After a 45-min equilibration period, the rings were contracted by the addition of KCl (40 mM). Concentration-response curves were obtained.

#### Culture of smooth muscle cells

VSMC derived from AVF were isolated from the mouse IVC. In brief, mouse IVC tissues were isolated and rinsed in cold PBS. The endothelial cells were removed by gently scraping the inner wall of the blood vessel with sterile ophthalmic forceps, and the adventitia was removed by peeling off the outer connective tissue of the blood vessel. All operations were performed in sterile ice-bathed PBS buffer to avoid tissue damage. After the endothelium and adventitia were removed, the tissues were cut into 1–2 mm pieces and treated with 0.25% collagenase type II and 0.5% elastase type II at 37 °C for 1 h. Mouse aortic smooth muscle cells were maintained in Dulbecco’s modified Eagle’s medium (DMEM)/F12 supplemented with 10% fetal bovine serum (FBS) and 1% penicillin/streptomycin and identified by immunofluorescence staining of α-smooth muscle actin (αSMA). Cells from passages 2–4 were used for further analysis. Human venous smooth muscle cells were purchased from ATCC with the Vascular Smooth Muscle Cell Growth Kit (ATCC PCS-100-04).

#### PCR and immunoblotting assays

Immunoblotting and real-time PCR assays were performed as previously described. Briefly, total protein lysates were extracted and resolved on SDS polyacrylamide electrophoresis gels and then transferred to polyvinylidene fluoride membranes. Antibody information is listed in the supplementary table 2. All assays were carried out using three independent biological replicates. Only one representative immunoblotting results from the three repeats is shown.

For real-time PCR assays, total cell RNA was extracted by TRIZOL reagent, and tissue RNA was isolated by mirVana miRNA isolation kit. All mRNA was treated with deoxyribonuclease to clean DNA resident. Reverse transcription was performed with superscript III (Invitrogen) and random hexamers according to the manufacture’s instruction. Real-time qPCR was run on the QuantStudio^™^ 7 system using the FastStart Universal SYBR Green Master mix. All real-time qPCR assays were performed in six technical replicates from three independent experiments. Primer information is listed in the supplementary table 3.

#### Cell contraction assays

Totally 1 mL of collagen I (2 mg/mL) and 0.4 mL of 5 × DMEM medium containing 0.2 mL of FBS (pH = 7.2) were mixed and kept on ice. Cells (1 × 10^5^/well) in 0.1 mL culturing medium were mixed with 0.4 mL gel solution by gently pipetting. The mixture was placed in 12-well tissue culture plates, incubated in 37 °C to allow polymerization, and added to 1 mL culturing medium. The gel matrix was gently detached from the well and scanned as time 0. The remaining area of gel was determined by Image J software. Gel contraction rate = (gel area after 24 h of culture/initial gel area) × 100%.

#### Cell proliferation and migration assays

Cell proliferation rates were measured by the MTS assays according to the manufacture’s protocol and were presented as relative fold change of absorbance at 450 nm and normalized to that at day zero.

The Bromodeoxyuridine (BrdU) label incorporation test was executed as per the guidelines provided by the manufacturer. In a summarized procedure, BrdU solution, diluted at 1:500, was administered to 1 × 10^4^ cells, which were then allowed to incubate for a duration of 16 h. Following this, BrdU incorporation was identified using an anti-BrdU antibody diluted at 1:1,000 and a horseradish peroxidase-conjugated secondary antibody. Subsequent to the addition of TMB peroxidase substrate, changes in absorbance at 450 nm were measured using a densitometer. The BrdU incorporation rate was expressed as a fold increase in OD450, standardized against the control sample.

#### MMPs activity assays

MMPs activity assays of VSMC were measured by the fluorometric-green MMP activity assay kit (ab112146, Abcam), in accordance with the manufacturer’s instructions.

### Co-immunoprecipitation

Cell lysates were extracted by NETN buffer (0.5% NP40, 1 mM of EDTA, 50 mM of Tris, and 150 mM of NaCl plus proteinase and phosphatase inhibitor). Pre-cleared lysates were incubated with IgG or indicated antibodies, and the associated proteins were immunoblotted by antibodies as indicated. Experiments were repeated at least three times, and one set of representative blots was shown.

#### Proximate ligation assay

Proximity ligation assay (PLA) assays were performed using the Duolink *in situ* Red Starter Kit Mouse/Rabbit (Sigma) as previously reported [17]. Cells were fixed with 4% paraformaldehyde and permeabilized by 0.2% Triton-X100. Fixed cells were incubated with primary antibodies overnight at 4 °C. Secondary probe, ligation, and amplification reactions were performed following the manufacturer’s instructions.

### Statistics

Results of tissues using a clinical or animal model were shown as biological replicates, and the cell experiments were shown as technical replicates. For cell experiments, all real-time PCR assays were carried out using six technical replicates and three independent cDNA syntheses. Western blotting assays were performed. Experiments were repeated in three independent experiments, and one of the representative blots was shown.

Data are represented as mean ± SEM. A Student’s *t*-test (two-sided) was used to compare two-group data with normal distribution and equivalent variance; for multiple groups with two independent factors, a one-way ANOVA with Tukey multiple comparisons was used for normally distributed variables. *p* < 0.05 was considered statistically significant. All date meet the assumptions of the tests. All statistical analyses were conducted on biological replicates in GraphPad Prism 9.0.

## Results

### Smooth muscle cells in the neointima of stenotic arteriovenous fistulae showed increased expression of PCSK6

**Figure 1.**
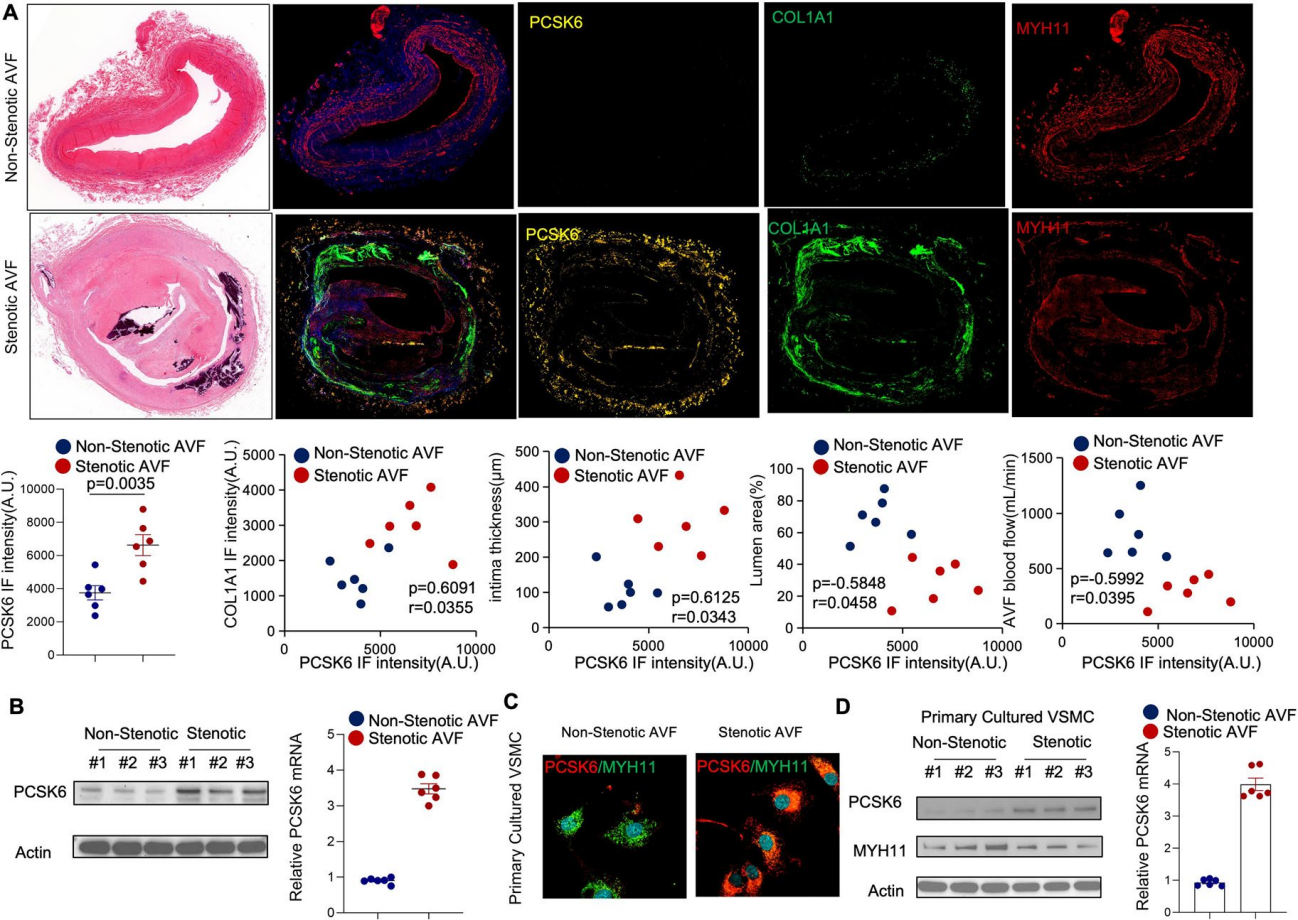
PCSK6 is increased in smooth muscle cells of stenotic arteriovenous fistula. (A-B) Human stenotic and non-stenotic arteriovenous fistula (AVF) tissues were obtained as described in the Materials and Methods. (A) Representative images of hematoxylin and eosin (HE) staining and immunofluorescence staining for PCSK6, COL1A1, and MYH11 in tissue sections. Immunofluorescence intensity of PCSK6 in the two groups, as well as correlations between PCSK6 and COL1A1 immunofluorescence intensity, neointimal thickness, degree of luminal stenosis, and AVF blood flow were plotted. (B) Total protein and RNA were extracted from tissues. Protein expression of PCSK6 was analyzed by Western blot, and mRNA expression was determined by real-time PCR. (C-D) Primary cultured smooth muscle cells (SMCs) were derived from human stenotic and non-stenotic AVF tissues. (C) Immunofluorescence staining for PCSK6 and the SMC marker MYH11 in primary cultured SMCs. (D) Total protein and RNA were extracted from primary cultured SMCs. Protein expression of PCSK6 was analyzed by Western blot, and mRNA expression was determined by real-time PCR.

To investigate the potential role of PCSK6 expression in intimal hyperplasia and lumen stenosis of AVF, we first obtained stenotic and non-stenotic AVF tissue samples and evaluated the expression levels of PCSK6, the collagen marker COL1A1, and the smooth muscle cell marker Myh11. The results demonstrated that PCSK6 expression was significantly upregulated in stenotic AVF tissues and correlated positively with COL1A1 expression. Furthermore, PCSK6 expression was associated with increased intimal thickness and negatively correlated with both lumen area and flow volume in AVF (Figure 1A). Consistent with these findings, Western blot and real-time PCR analyses confirmed elevated PCSK6 expression in stenotic AVF tissues (Figure 1B).

Given previous evidence indicating that dedifferentiation of VSMCs significantly contributes to neointimal hyperplasia and subsequent AVF stenosis, we sought to determine whether increased PCSK6 expression occurs specifically in VSMCs. Immunofluorescence co-staining of cells isolated from stenotic and non-stenotic AVF tissues revealed enhanced PCSK6 expression in MYH11-positive cells (Figure 1C). This observation was further supported by Western blot and real-time PCR data that showed that PCSK6 was increased in primary cultured VSMCs derived form stenotic AVF tissues (Figure 1D). Given our clinical findings linking PCSK6 upregulation to AVF stenosis, we next evaluated the impact of PCSK6 expression on AVF patency and post-failure intervention burden. Kaplan-Meier survival analysis demonstrated that patients with high PCSK6 expression exhibited significantly shorter AVF patency time compared to those with low PCSK6 expression (Figure S2C). Collectively, these findings suggest that PCSK6 may exert regulatory effects on intimal hyperplasia and stenosis in human AVF.

### PCSK6 was induced in SMCs during intimal hyperplasia formation

**Figure 2.**
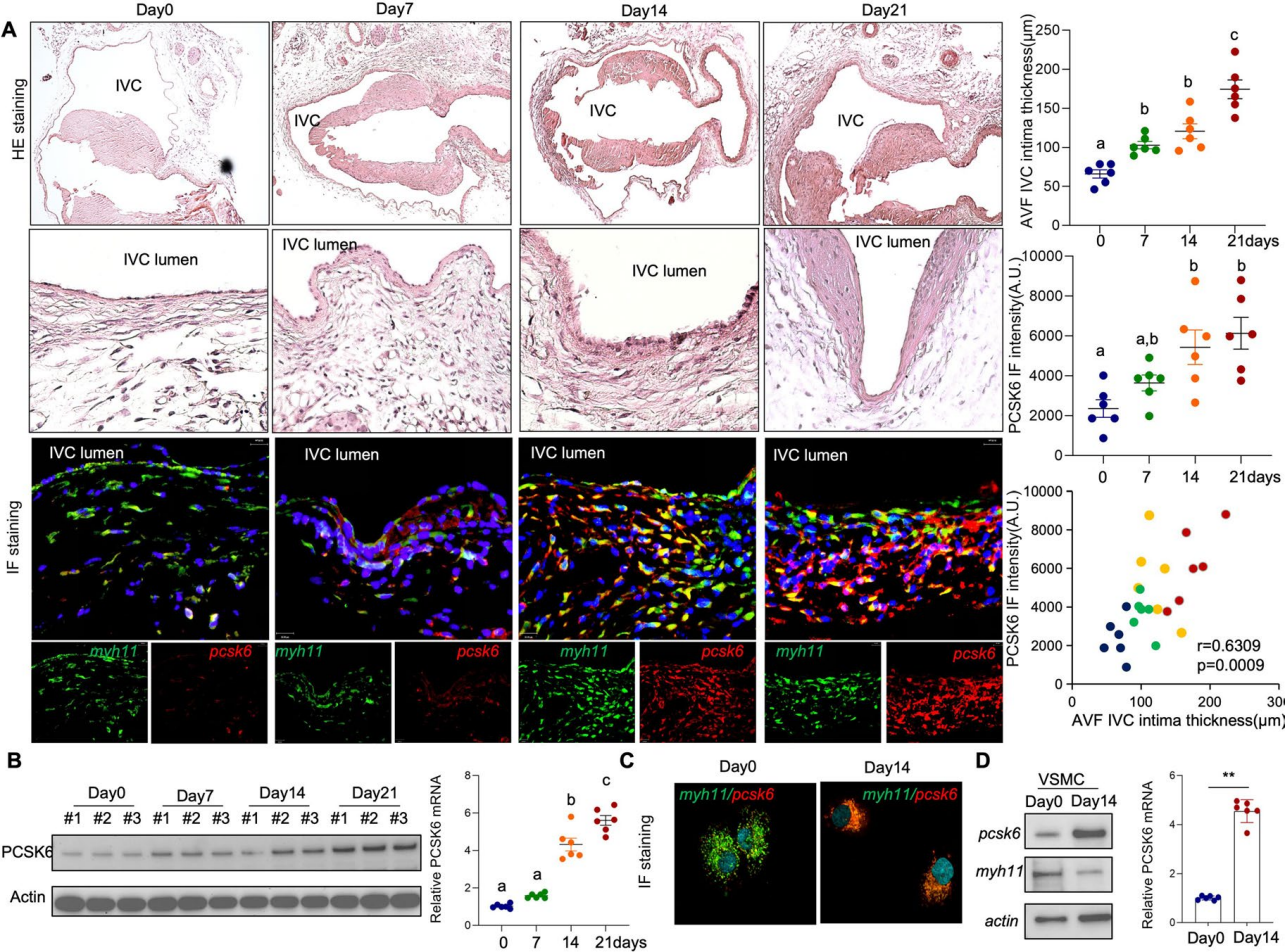
PCSK6 is increased in smooth muscle cells during venous remodeling after arteriovenous creation. (A-B) Mouse AVF models were generated as described in the Material and Methods. (A) Tissues from the AVF anastomosis were collected at the indicated time points. Representative images of HE staining and immunofluorescence staining for PCSK6 and MYH11 are shown. Neointimal thickness and PCSK6 immunofluorescence intensity across different time points, as well as the correlation between PCSK6 intensity and neointimal thickness were plotted. (B) Total protein and RNA were extracted from the tissues. Protein expression of PCSK6 at different time points was analyzed by Western blot, and mRNA expression was determined by real-time PCR. (C-D) Primary cultured SMCs were derived from the AVF at the indicated time points. (C) Immunofluorescence staining for PCSK6 and the SMC marker MYH11 in primary cultured SMCs. (D) Total protein and RNA were extracted from primary cultured SMCs. Protein expression of PCSK6 was analyzed by Western blot, and mRNA expression was determined by real-time PCR.

Next, to further evaluate the potential association between PCSK6 expression and intimal hyperplasia in AVF, we utilized a mouse AVF model for detailed longitudinal analysis (Figure S2A). Histological assessment combined with systematic morphometric analysis indicated a progressive increase in the severity of intimal hyperplasia over time following AVF creation. Immunofluorescence staining further demonstrated a corresponding increase in PCSK6 expression within the intimal tissues of the mouse AVF. Quantitative analysis confirmed that the upregulation of PCSK6 was significantly correlated with the extent of neointimal thickening (Figures 2A and S2B). This pattern of elevated PCSK6 expression during neointimal progression was consistently observed at the protein and transcriptional levels, as validated by Western blot and real-time PCR assays (Figure 2B). In addition, analysis of primary cultured VSMCs isolated from the AVF model showed a time-dependent increase in PCSK6 expression post-AVF surgery, which supported the *in vivo* findings (Figure 2C and D). Collectively, these data align with the expression profile observed in human AVF samples and support the hypothesis that PCSK6 overexpression may be functionally implicated in the mechanisms driving neointimal hyperplasia in AVF.

### PCSK6 promotes SMCs activation and ECM remodeling

**Figure 3.**
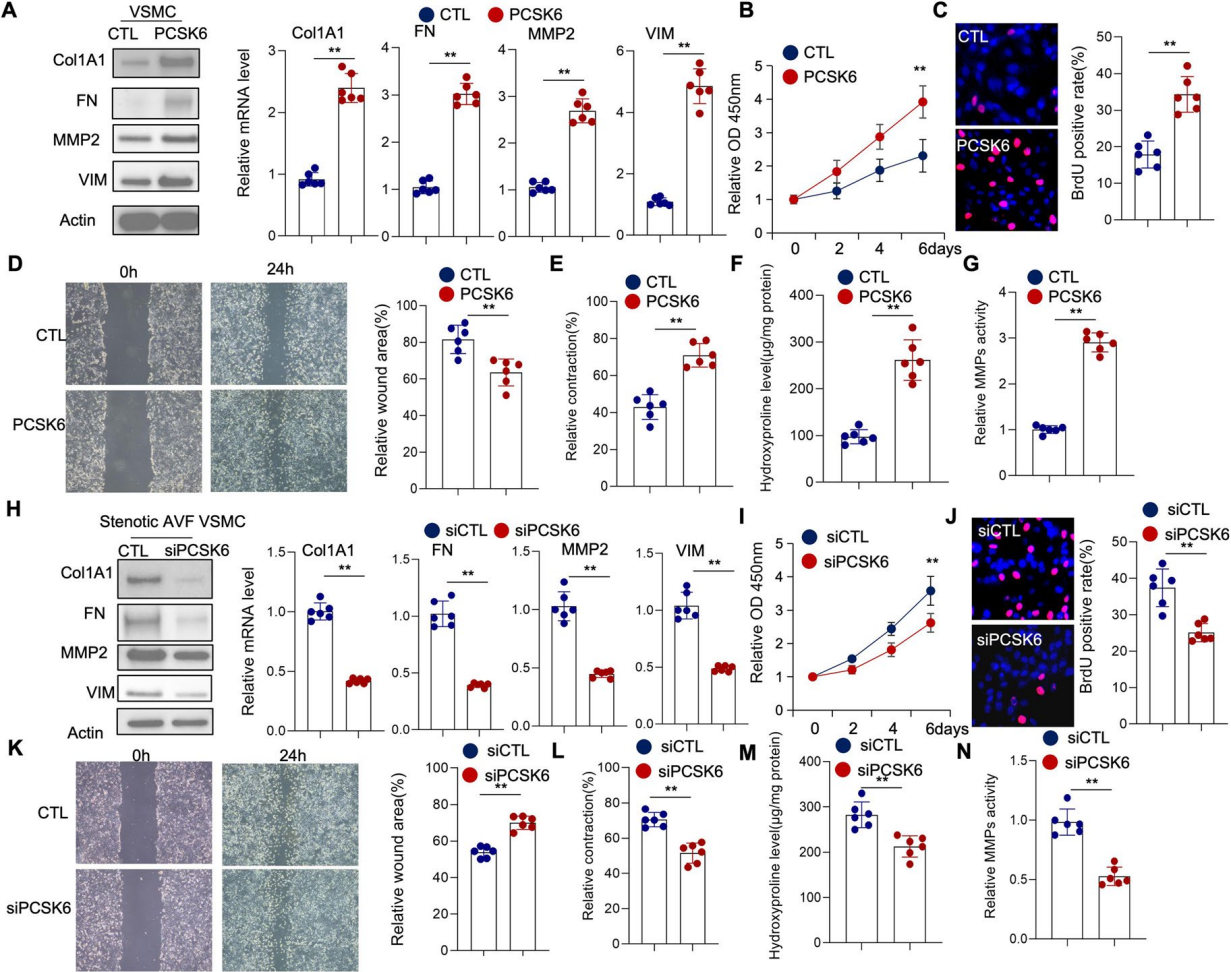
PCSK6 promotes smooth muscle cells phenotypic switch and ECM production. (A–G) Venous SMCs were transfected with control or PCSK6 expression vectors. (A) Total protein and RNA were extracted. Protein expression of COL1A1, fibronectin, VIM, and MMP2 was analyzed by Western blot, and mRNA expression was determined by real-time PCR. (B) Cell viability was assessed using CCK-8 assay. (C) Cell proliferation was measured by BrdU assay. (D) Cell migration was evaluated by wound healing assay. (E) Cell contractility was determined by collagen gel contraction assay. (F). Hydroxyproline levels were quantified. (G) MMPs activity was measured using MMPs activity kit as described in the Material and Methods section. (H–N) PrimaryM cultured SMCs were transfected with siRNA targeting either control or PCSK6. (H) Total protein and RNA were extracted. Protein expression of COL1A1, fibronectin, VIM, and MMP2 was analyzed by Western blot, and mRNA expression was determined by real-time PCR. (I) Cell viability was assessed using CCK-8 assay. (J) Cell proliferation was measured by BrdU assay. (K) Cell migration was evaluated by wound healing assay. (L) Cell contractility was determined by collagen gel contraction assay. (M) Hydroxyproline levels were quantified. (N) MMPs activity was measured using MMPs activity kit as described in the Material and Methods section.

Although the aforementioned findings strongly suggest an involvement of PCSK6 in the processes of intimal hyperplasia and AVF stenosis, the precise molecular and cellular mechanisms underlying this effect remained to be elucidated. We therefore postulated that PCSK6 contributes to the development of neointimal lesions primarily through the regulation of VSMC phenotype and function. To investigate this hypothesis, we first overexpressed PCSK6 in cultured VSMCs and analyzed the resulting phenotypic changes. This manipulation led to a significant increase in the expression of key synthetic-state markers, including VIM, COL1A1, fibronectin, and MMP2 (Figure 3A). Functional assays further demonstrated that PCSK6 overexpression robustly stimulated VSMC proliferation, as quantitatively assessed by both CCK-8 assay (Figure 3B) and BrdU incorporation assay (Figure 3C). In addition, scratch wound healing assays performed on these cells revealed a pronounced enhancement of their migratory capacity following PCSK6 overexpression (Figure 3D). The functional impact extended to contractile properties, where collagen gel contraction assays indicated that PCSK6 also potentiates VSMC-mediated contraction (Figure 3E). To explore the role of PCSK6 in extracellular matrix (ECM) remodeling, a critical component of neointimal formation, we conducted hydroxyproline assays to quantify collagen production and zymography to assess MMP activity. The results confirmed that PCSK6 overexpression in VSMCs significantly promotes both collagen synthesis and matrix protease activity (Figure 3F and G).

To substantiate the specificity of these observations, we performed loss-of-function experiments by knocking down PCSK6 expression in primary VSMCs isolated from human stenotic AVF tissues. The knockdown effectively attenuated the expression of the synthetic markers (Figure 3H). Concomitantly, it led to a significant suppression of multiple VSMC functions, including proliferation, migration, contractility, and the synthesis of collagen and matrix proteases (Figure 3I–N). Taken together, these comprehensive gain- and loss-of-function analyses consistently demonstrate that PCSK6 plays a critical role in promoting the activated synthetic phenotype of VSMCs and in driving ECM remodeling, thereby identifying a likely mechanism by which PCSK6 contributes to intimal hyperplasia in AVF.

### Suppressing STAT1 antagonized the effect of PCSK6 on SMCs proliferation, migration and ECM remodeling

**Figure 4.**
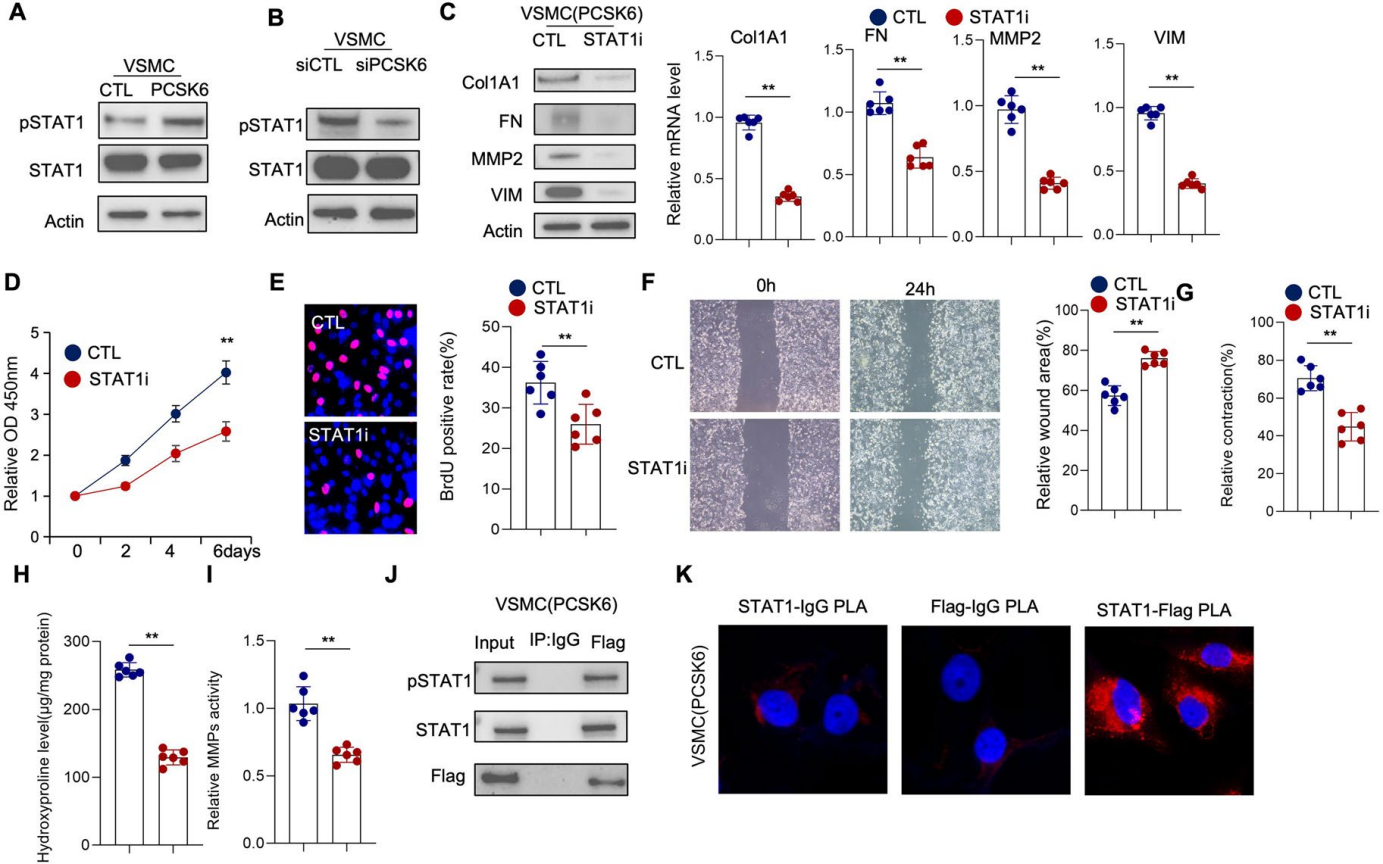
Suppressing STAT1 antagonized the effect of PCSK6, alleviated VSMCs phenotypic switch and ECM production. (A) SMCs were transfected with control or Flag-PCSK6 expression vectors. Total protein was extracted and analyzed by Western blotting for total STAT1 and phosphorylated STAT1 levels. (B) SMCs were transfected with control siRNA or siRNA targeting PCSK6. Total protein was extracted and analyzed by Western blotting for total STAT1 and phosphorylated STAT1 levels. (C–I) SMCs overexpressing PCSK6 were treated with control or the STAT1 inhibitor fludarabine. (C) Total protein and RNA were extracted. Protein expression of COL1A1, fibronectin, VIM, and MMP2 was analyzed by Western blot, and mRNA expression was determined by real-time PCR. (D) Cell viability was assessed by CCK-8 assay. (E) Cell proliferation was measured by BrdU assay. (F) Cell migration was evaluated by wound healing assay. (G) Hydroxyproline levels were quantified. (H) Cell contractility was determined by collagen gel contraction assay. (I) MMP activity was measured by MMP activity assay. (J) Total protein was extracted from SMCs overexpressing Flag-PCSK6. Co-IP was performed using IgG or Flag antibody, and the immunoprecipitated proteins were detected with antibodies against STAT1 and phosphorylated STAT1. (K) PLA assays was performed in SMCs overexpressing Flag-PCSK6 using Flag and STAT1 antibodies. Representative PLA images are shown.

Based on previous studies implicating STAT1 in the regulation of SMC phenotypic switching and extracellular matrix synthesis [18], we sought to investigate its potential involvement in PCSK6-mediated signaling. In VSMCs overexpressing PCSK6, we observed a significant increase in STAT1 phosphorylation levels (Figures 4A and S3A). Conversely, knockdown of PCSK6 in primary cultured VSMCs isolated from stenotic AVF tissues resulted in a marked reduction in STAT1 phosphorylation (Figures 4B and S3B), collectively suggesting that PCSK6 activates STAT1 signaling in VSMCs. We evaluated the protein and mRNA levels of genes related to extracellular matrix (ECM) and phenotypic transition in vascular smooth muscle cells (VSMCs) overexpressing PCSK6. Both pharmacological inhibition (fludarabine, STAT1i) and siRNA-mediated knockdown of STAT1 significantly weakened the upregulation of Col1A1, FN, MMP2, and VIM mRNA driven by PCSK6 overexpression (Figures 4C and S3C, S5A). To further determine whether STAT1 activation mediates the functional effects of PCSK6 on SMC activation and ECM remodeling, we treated PCSK6-overexpressing VSMCs with the STAT1 inhibitor fludarabine and additionally performed siRNA-mediated STAT1 knockdown in VSMCs for genetic validation. Pharmacological inhibition of STAT1 significantly attenuated the PCSK6-induced enhancement of VSMC proliferation, migration, and contractile function (Figure 4D–G). Consistently, siRNA-mediated silencing of STAT1 also markedly suppressed VSMC proliferation (Figure S5B, C), migration (Figure S5D), and contractile capacity (Figure S5E). Furthermore, both fludarabine treatment and STAT1 knockdown suppressed the PCSK6-driven increases in collagen synthesis and MMP production (Figures 4H, I and S5F, G). These results indicate that PCSK6 promotes SMC activation primarily through STAT1-dependent mechanisms.

Given previous bioinformatic predictions suggesting a potential interaction between PCSK6 and STAT1 [19], we performed co-immunoprecipitation experiments in PCSK6-overexpressing VSMCs. Results demonstrated a direct physical association between PCSK6 and STAT1 (Figure 4J). This interaction was further confirmed through proximity ligation assay (PLA), which revealed significant co-localization of PCSK6 and STAT1 in the cellular context (Figure 4K). Together, these findings demonstrate that PCSK6 physically interacts with and activates STAT1, thereby promoting SMC phenotypic transition and ECM synthesis.

### SMC-specific knockout of PCSK6 attenuated intimal hyperplasia and improved fistula patency

**Figure 5.**
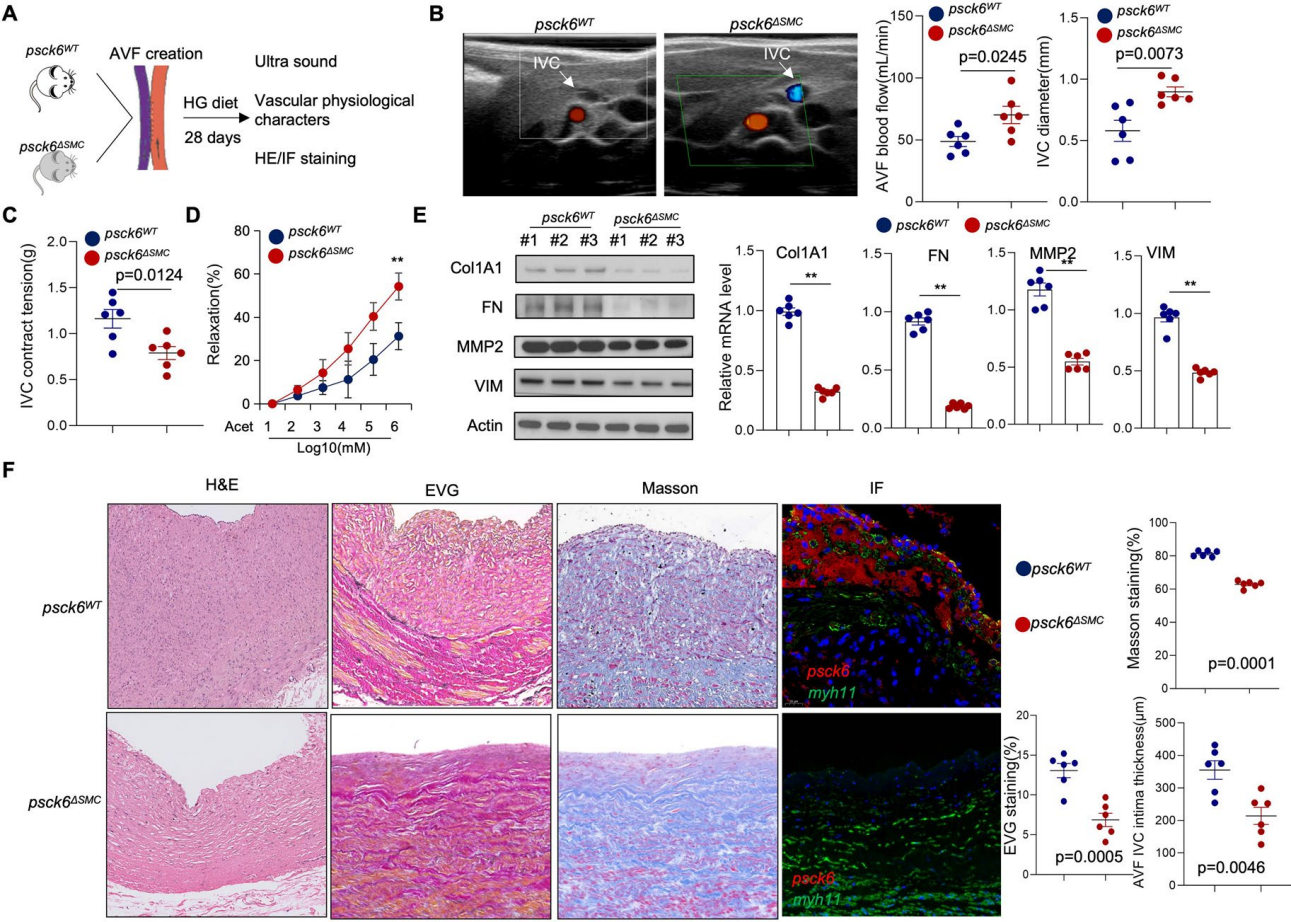
Silencing of PCSK6 in VSMCs alleviated venous remodeling and AVF stenosis. (A) Smooth muscle cell-specific PCSK6 knockout mice were generated as described in the Material and Methods. The schematic illustrates the experimental timeline after AVF creation in both knockout and control mice. (B) AVF diameter and blood flow were monitored by ultrasound. Quantitative data are presented. (C-D) Functional analysis of harvested IVC segments assessing (C) contraction responses to 40mM KCl and (D) Relaxation responses to the cumulative addition of acetylcholine. (E) Total protein and RNA were extracted. Protein expression of COL1A1, fibronectin, MMP2 and VIM, was analyzed by Western blot, and mRNA expression was determined by real-time PCR. (F) Histological evaluation of IVC sections through HE/EVG/Masson staining and immunofluorescence for PCSK6 and MYH11. Neointimal thickness was quantified in both experimental groups.

To further validate the physiological significance of PCSK6-mediated smooth muscle cell activation observed in our *in vitro* experiments, we established a smooth muscle cell-specific PCSK6 knockout mouse model (*pcsk6^ΔSMC^*) to investigate its functional impact on AVF maturation and stenosis development *in vivo*. Comprehensive histological evaluation confirmed the absence of preexisting arterial or venous abnormalities or compromised vascular development in *pcsk6^ΔSMC^* prior to AVF creation (Figure S4A). Following AVF creation, both *pcsk6^ΔSMC^* and control littermates (*pcsk6^WT^*) were maintained on a high-glucose diet to promote stenosis development (Figure 5A). Longitudinal monitoring using Doppler ultrasound demonstrated that *pcsk6^ΔSMC^* maintained significantly larger AVF lumen diameters and exhibited superior blood flow parameters throughout the observation period (Figure 5B). The specific time points of longitudinal monitoring are 1 week, 2 weeks, and 4 weeks postoperatively; the results shown in the illustration were measured in the fourth week. Importantly, echocardiographic analysis confirmed comparable left ventricular ejection fractions between *pcsk6^ΔSMC^* and *pcsk6^WT^* mice (Figure S4B), effectively excluding potential confounding effects of differential cardiac performance on the observed vascular phenotypes.

Detailed vascular functional characterization revealed substantial alterations in vasoreactivity following PCSK6 deletion. Specifically, maximum contractile responses to KCl (40 mM) stimulation were markedly attenuated in vessels from *pcsk6^ΔSMC^* (Figure 5C). Complementing this finding, endothelium-dependent vasodilation in response to acetylcholine was significantly enhanced in the *pcsk6^ΔSMC^* group (Figure 5D). These coordinated changes in vascular tone regulation collectively indicate that SMC-specific PCSK6 deletion creates a vascular environment favoring dilation over constriction, thereby facilitating improved AVF hemodynamics.

Consistent with these functional improvements, detailed histomorphometry and immunofluorescence analyses at the endpoint examination demonstrated a pronounced reduction in neointimal hyperplasia within the AVF of *pcsk6^ΔSMC^*. The attenuated neointimal formation was accompanied by significantly improved lumen patency in *pcsk6^ΔSMC^* mice compared to controls (Figure 5E and F). Taken together, these *in vivo* findings provide compelling evidence that SMC-specific PCSK6 deletion effectively limits the development of neointimal hyperplasia and lumen stenosis, thereby enhancing overall AVF patency rates in the mouse model.

## Disscussion

In our research, we have identified PCSK6 as a critical regulator of venous neointimal hyperplasia, demonstrating its specific upregulation in VSMCs of both human and murine stenotic AVFs. This elevated expression correlates strongly with the severity of neointimal formation and lumen stenosis, thereby establishing our findings as an important extension of PCSK6 biology to the AVF setting.

Our findings substantially extend the current understanding of PCSK family functions in vascular pathology. Previous research has primarily focused on PCSK9 in lipid metabolism and artery neointima formation [20,21]. While PCSK6 has been implicated in atherosclerosis and kidney diseases [22], this study establishes a distinct role of PCSK6 in AVF failure. The *in vivo* validation using smooth muscle-specific PCSK6 knockout mice offers compelling evidence for the therapeutic potential of targeting this pathway. The significant attenuation of neointimal hyperplasia, coupled with improved AVF lumen diameter and blood flow in knockout animals, underscores the central role of PCSK6 in AVF remodeling. Notably, these beneficial effects occurred without compromising cardiac function, suggesting a favorable safety profile for therapeutic interventions aimed at this pathway.

From a clinical perspective, although our study confirmed a strong correlation between PCSK6 expression levels and the degree of stenosis in human arteriovenous fistula samples, and we effectively reversed PCSK6-mediated vascular smooth muscle cell activation through pharmacological inhibition of STAT1, further preclinical studies in more complex models and validation in larger, prospective clinical cohorts are necessary to establish PCSK6 as a viable biomarker or therapeutic target for AVF stenosis.

Our human venous samples were obtained from patients who had received kidney transplants. While this cohort provided access to tissue from definitively failed AVFs, it may introduce a selection bias toward patients who were clinically stable enough for transplantation. The generalizability of our findings to the broader dialysis population should be confirmed in future studies utilizing tissues from non-transplanted patients.

The clinical sample size of this study was 6 cases per group. Although there were no significant differences in baseline data, the small sample size may reduce the statistical test power. Meanwhile, we analyzed the selection bias: all enrolled samples were patients after kidney transplantation. In the follow-up study, we will expand the sample size and include non-transplant maintenance hemodialysis patients to further verify the role of PCSK6 in AVF stenosis. In addition, this study has verified the core conclusions through multiple levels of animal experiments and *in vitro* cell experiments, which makes up for the deficiency of clinical sample size to a certain extent.

Several aspects of PCSK6 regulation and function merit further investigation. The upstream triggers for PCSK6 upregulation following AVF creation, particularly the specific hemodynamic forces and molecular signals involved, remain to be fully characterized. Additionally, potential contributions from other cell types, including endothelial cells and inflammatory cells, to PCSK6-mediated remodeling represent important areas for future research.

This study adopted 9–12-week-old young male C57BL/6 mice without considering the influence of age and gender, which is an important limitation of this study. In the follow-up, we will construct AVF models of different ages (elderly mice) and male-female pairing to further verify the role of the PCSK6/STAT1 axis. Meanwhile, the model results of young male mice provide core mechanism evidence for basic research and lay a foundation for subsequent clinical translational research.

In conclusion, this study establishes the PCSK6/STAT1 axis as a novel regulatory mechanism driving venous neointimal hyperplasia in AVF remodeling. These findings not only advance our understanding of the molecular basis of AVF failure but also identify new diagnostic and therapeutic opportunities for improving vascular access outcomes in hemodialysis patients.

## Supplementary Material

PCSK6 AVF S3.jpg

PCSK6 AVF S4.jpg

PCSK6 AVF S5.jpg

PCSK6 AVF S1.jpg

PCSK6 AVF S2.jpg

## Data Availability

The data that support the findings of this study are available from the corresponding author upon reasonable request.
