# Supplementary figures and images for: PCSK6 is a novel regulator of venous smooth muscle cell function in arteriovenous fistula remodeling
Source: Ren Fail. 2026 May 5;48(1):2663246. doi: 10.1080/0886022X.2026.2663246 (PMC13148085; doi:10.1080/0886022X.2026.2663246)

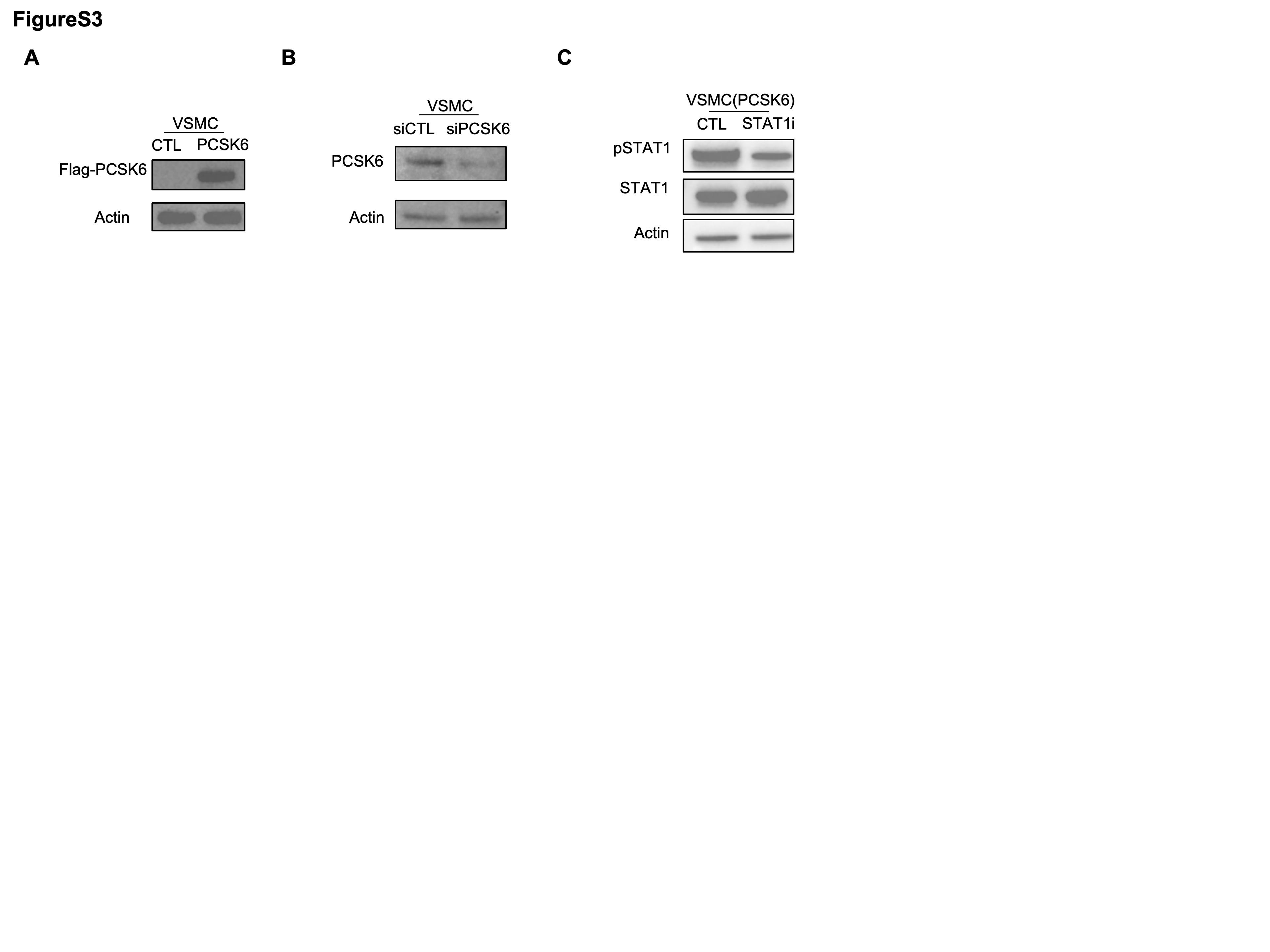

Supplement: PCSK6 AVF S3.jpg [file IRNF_A_2663246_SM7378.jpg]

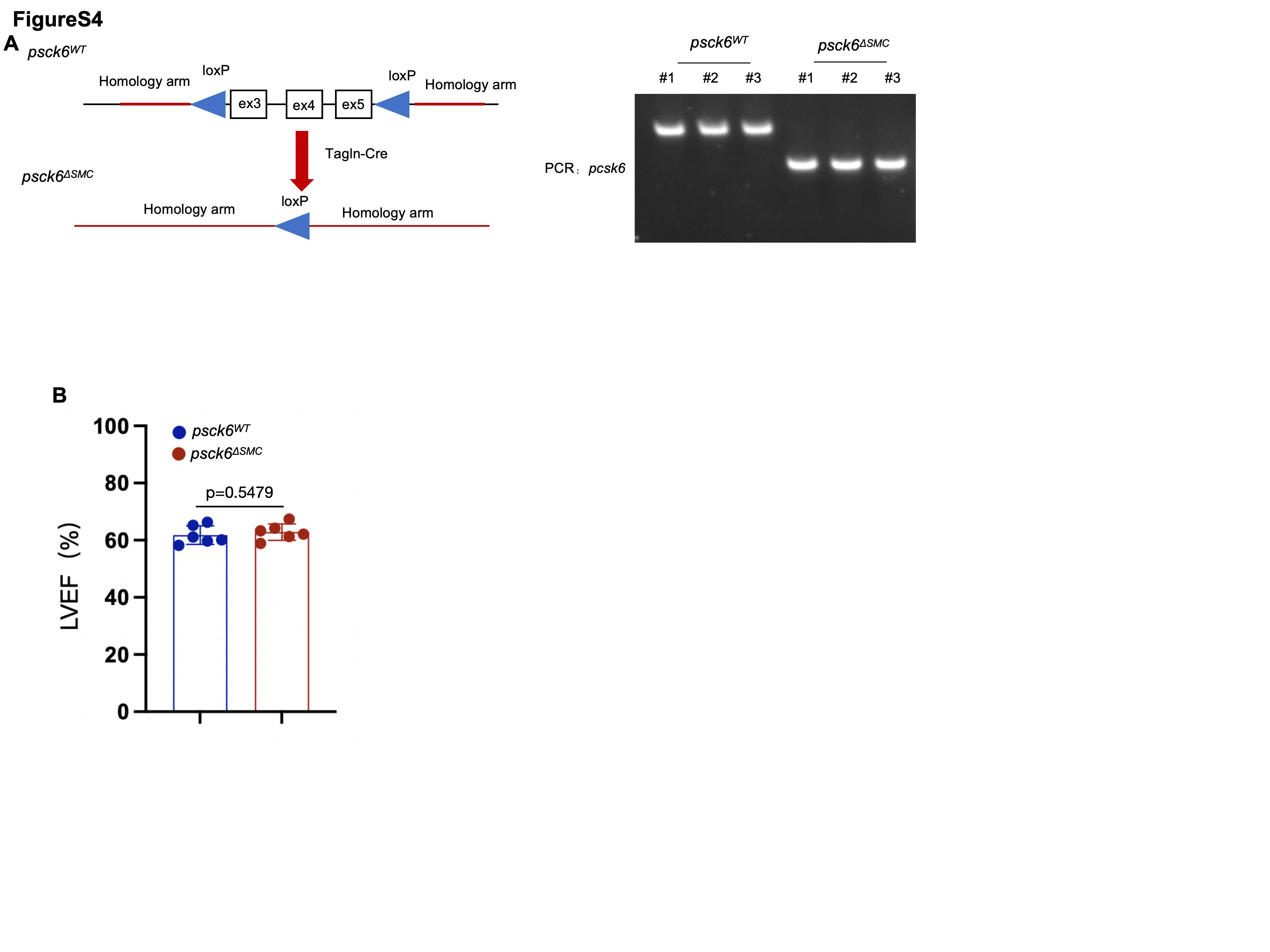

Supplement: PCSK6 AVF S4.jpg [file IRNF_A_2663246_SM7377.jpg]

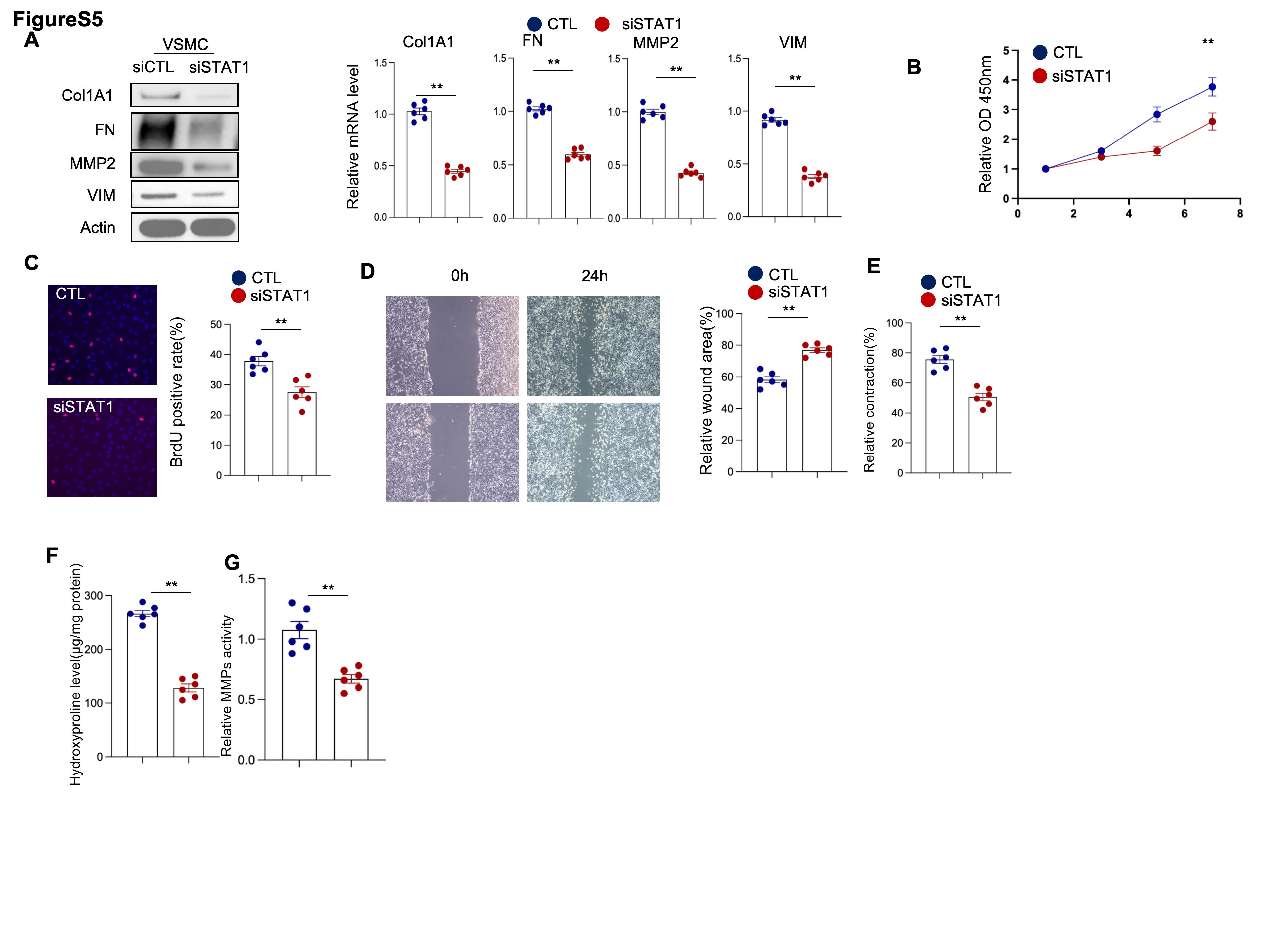

Supplement: PCSK6 AVF S5.jpg [file IRNF_A_2663246_SM7376.jpg]

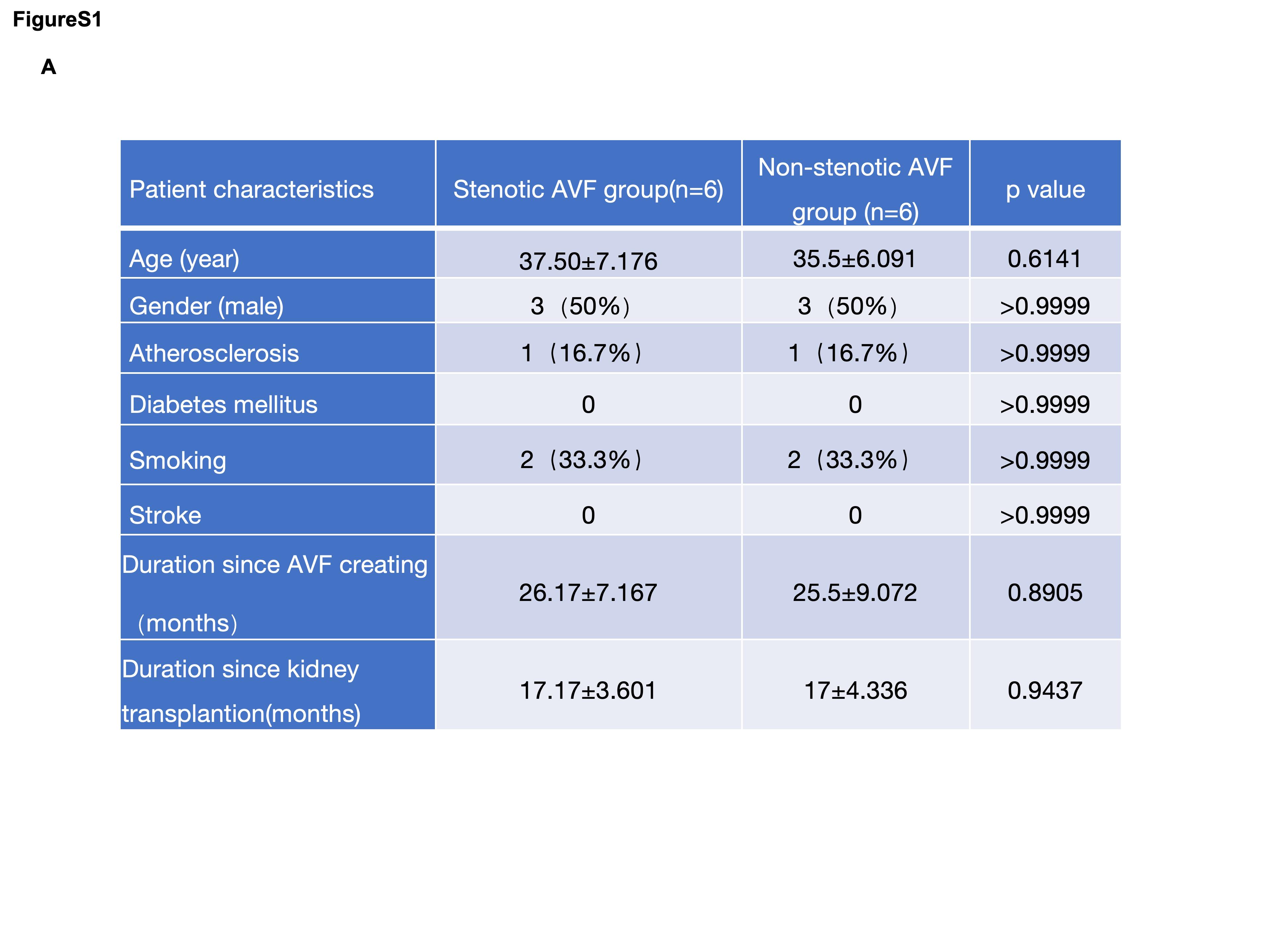

Supplement: PCSK6 AVF S1.jpg [file IRNF_A_2663246_SM7375.jpg]

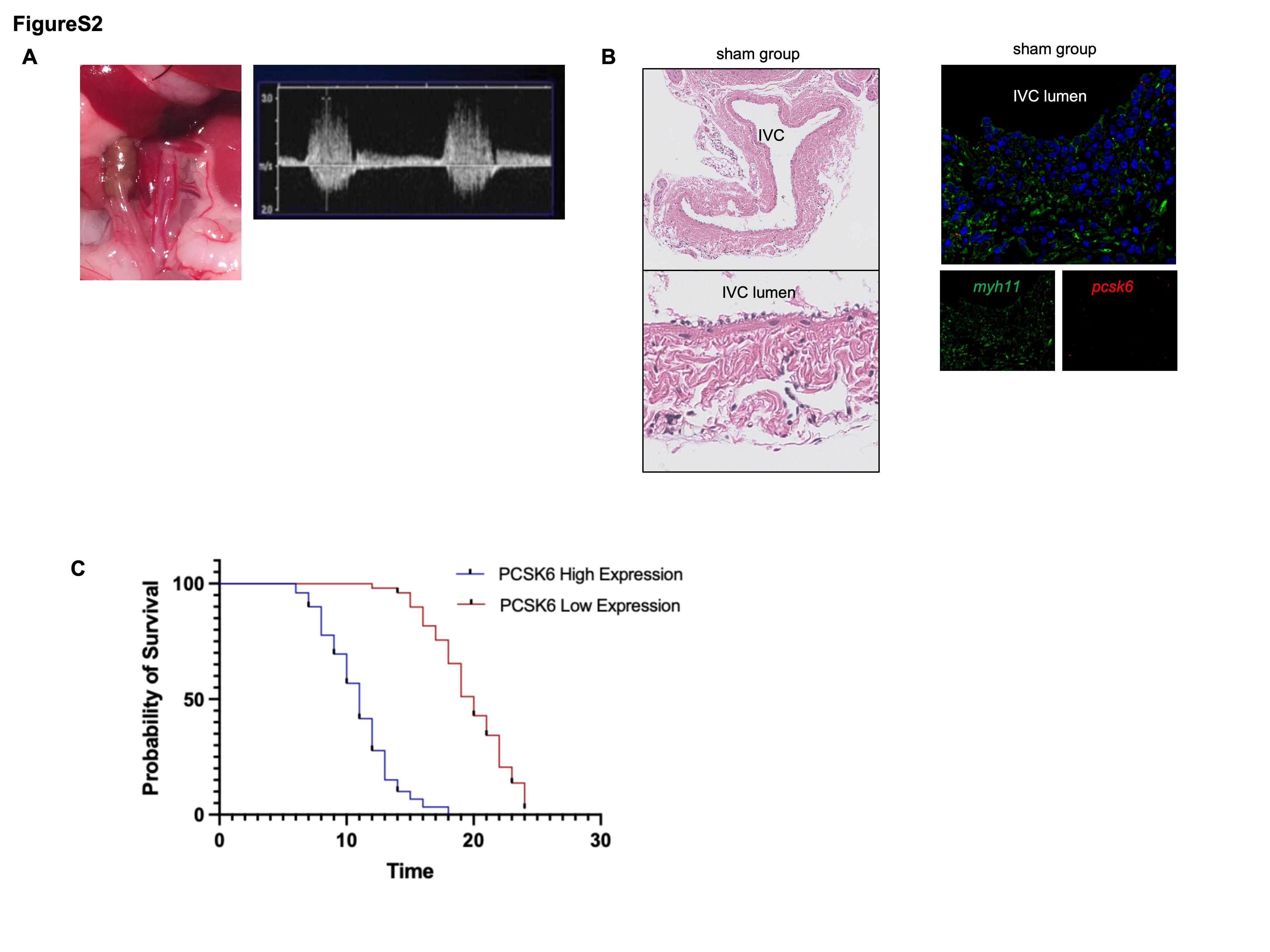

Supplement: PCSK6 AVF S2.jpg [file IRNF_A_2663246_SM7373.jpg]
